# Developing a multiscale, multi-resolution agent-based brain tumor model by graphics processing units

**DOI:** 10.1186/1742-4682-8-46

**Published:** 2011-12-16

**Authors:** Le Zhang, Beini Jiang, Yukun Wu, Costas Strouthos, Phillip Zhe Sun, Jing Su, Xiaobo Zhou

**Affiliations:** 1 Department of Mathematical Sciences, Michigan Technological University, Houghton, MI, 49931, USA; 2 College of Computer and Information Science, Southwest University, Chongqing, 400715, China; 3 Center for Vaccine Development, University of Maryland School of Medicine, Baltimore, MD 21201, USA; 4 Computation-based Science and Technology Research Center, The Cyprus Institute, 1645 Nicosia, Cyprus; 5 Harvard-MIT (HST) Athinoula A. Martinos Center for Biomedical Imaging, Massachusetts General Hospital, Charlestown, MA, USA; 6 Department of Pathology, the Methodist Hospital, Research Institute & Weill Cornell Medical College, 6565 Fannin St, Houston, Texas, USA

## Abstract

Multiscale agent-based modeling (*MABM*) has been widely used to simulate Glioblastoma Multiforme (*GBM*) and its progression. At the intracellular level, the *MABM *approach employs a system of ordinary differential equations to describe quantitatively specific intracellular molecular pathways that determine phenotypic switches among cells (e.g. from migration to proliferation and vice versa). At the intercellular level, *MABM *describes cell-cell interactions by a discrete module. At the tissue level, partial differential equations are employed to model the diffusion of chemoattractants, which are the input factors of the intracellular molecular pathway. Moreover, multiscale analysis makes it possible to explore the molecules that play important roles in determining the cellular phenotypic switches that in turn drive the whole *GBM *expansion. However, owing to limited computational resources, *MABM *is currently a theoretical biological model that uses relatively coarse grids to simulate a few cancer cells in a small slice of brain cancer tissue. In order to improve this theoretical model to simulate and predict actual *GBM *cancer progression in real time, a graphics processing unit (*GPU*)-based parallel computing algorithm was developed and combined with the multi-resolution design to speed up the *MABM*. The simulated results demonstrated that the *GPU*-based, multi-resolution and multiscale approach can accelerate the previous *MABM *around 30-fold with relatively fine grids in a large extracellular matrix. Therefore, the new model has great potential for simulating and predicting real-time *GBM *progression, if real experimental data are incorporated.

## Background

Glioblastoma multiforme (*GBM*) is the most common and aggressive brain cancer [1,2]. Statistics show that it has the worst prognosis of all central nervous system malignancies [3,4]. However, with the resolution of functional magnetic resonance imaging (*fMRI*) [5,6], currently limited to around 2-3 mm, even the most experienced clinical personnel cannot accurately forecast *GBM *progression. The difficulties of making such forecasts motivated computational biologists to develop multiscale mathematical models to explore the expansion and invasion of *GBM *[7-9].

Cancer behaves as a complex, dynamic, adaptive and self-organizing system [10], and agent-based models (*ABM*) are capable of describing such a system as a collection of autonomous and decision-making agents, which represent the cells. Therefore, computational biologists hope that with the *ABM *approach they can surpass the current limitations of imaging technology and predict tumor progression [11-16]. Our previous studies [15,16] developed various multiscale *ABMs *to simulate *GBM *progression. In these models, a cell's intracellular epidermal growth factor receptor (*EGFR*) signaling pathway is stimulated by a chemoattractant (such as transforming growth factor *α *(*TGFα*)), which diffuses at the tissue level. We also assumed that the transient rate of change of phospholipase *Cγ *(*PLCγ *), an important molecule in the *EGFR *pathway, will result in cancer cell migration, whereas a smooth rate of change of *PLCγ *will result in cancer cell proliferation [11,12,15,16]. At the intercellular scale, the behaviors of cells (such as the autocrine or paracrine secretion of chemoattractants and migration or proliferation phenotypes) remodel the tumor microenvironment and affect the overall tumor dynamics at the tissue level.

An important advantage of multiscale agent-based modeling (*MABM*) [15,16] is that we can employ multiscale analysis to investigate the incoherent connections among various scales. For example, we can depict the intracellular (molecular) profiles that lead to phenotypic switches at any cell's dynamic cross points (migration cell number crosses with proliferation cell number) [15] or in the interesting tumor regions [16]. Thus, *MABM *models [15-17] can be used as tools for generating experimentally testable hypotheses. The consequent validation experiments may reveal potential therapeutic targets.

Though *MABM *approaches have a great potential for investigating *GBM *progression, their complexity necessitates immense computational resources [15,17], which becomes forbidding for real-time simulations of spatio-temporal *GBM *progression. In fact, two problems prevent *MABM *doing real-time simulation. The first is that the computation time required for intracellular pathway computing for cancer cells will become huge, since a real cancer system may consist of millions of cells. The second is that it is impossible to employ a conventional sequential numerical solver to model the real-time diffusion of chemoattractants in a large extracellular matrix (*ECM*) with relatively fine grids.

To overcome the computation time problems, this study incorporates a graphics processing unit (*GPU*)-based parallel computing algorithm [18] into a multi-resolution design [16] to speed up the previous *MABM *[15,17]. The multi-resolution design [16] classified the cancer cells into heterogeneous and homogeneous clusters. The heterogeneous clusters consisted of migrating and proliferating cancer cells in the region of interest, whereas the homogeneous clusters comprised dead or quiescent cells. The limited computational resource was concentrated on the heterogeneous clusters to investigate the molecular profiles of migrating and proliferating cancer cells, while the quiescent and dead cells in the homogeneous clusters were treated with less of the resource. The *GPU*-based parallel computing algorithm can not only model the diffusion of chemoattractants in a large *ECM *with relatively fine grids in real time, but also process computing queries concerning the intracellular signaling pathways of millions of cancer cells in a real cancer progression system.

The results presented in this paper demonstrate that the *GPU*-based multi-resolution *MABM *has certain novel features that can help cancer scientists to explore the mechanism of *GBM *cancer progression. First, it is able to simulate real-time cancer progression in a large *ECM *with relatively fine grids. Second, since multiscale analysis [15,17] can reveal the correlations between *GBM *tumor progression and molecular concentration changes, we can tell which molecular species are the important biomarkers that impact tumor progression. Third, a multi-resolution design [16] not only allows us to visualize cancer progression by displaying all the cancer cell clusters in the tissue, but also enables us to track each cancer cell's trajectory.

In the following sections, we will introduce the previously-developed multiscale and multi-resolution *ABM*, describe how to use *GPU *to accelerate the simulation of the model, and finally illustrate the advantages of the model that can be used to analyze important biomarkers to inhibit *GBM *expansion and predict *GBM *progression.

## Mathematical model

### Multiscale perspective

**Figure 1:**
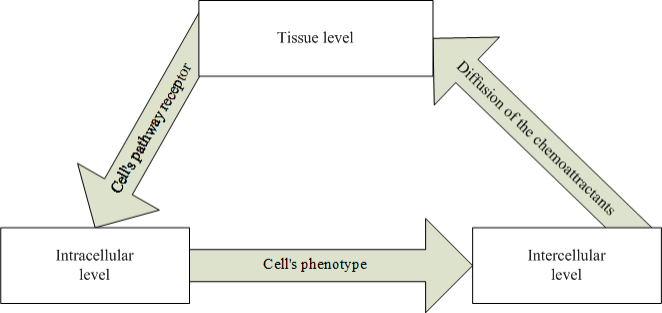
**Multiscale model of GBM growth**.

The multiscale approach was incorporated into *ABM*s to simulate *GBM *progression by incorporating into the model the interactions between different scales - the intracellular (gene-protein interaction) and the cellular (including cell-cell interactions and phenotypic switches e.g. from migration to proliferation and vice versa) - which in turn affect the spatio-temporal evolution of *GBM *(tissue scale). The relationships among the intracellular, cellular and tissue scales were conceptually defined as "interfaces". As indicated in Figure 1: (a) A cell's phenotype is defined as an interface between the intracellular and intercellular levels. The signaling pathway at intracellular level determines the cell's phenotype, which regulates intercellular behaviors. (b) We denote the diffusion of chemoattractants as an interface between the intercellular and tissue levels. At the tissue level, cells secrete chemoattractants that diffuse according to their concentration gradients and remodel the microenvironment of the tumor. (c) A cell's pathway receptors are defined as an interface between tissue and intracellular level. The paracrine or autocrine effects of chemoattractants on the tissue level are sensed by cellular receptors and trigger intracellular signaling pathways to determine a cell's phenotype.

### Intracellular scale

**Figure 2:**
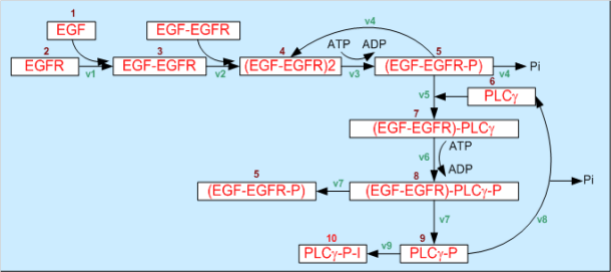
**Intracellular *EGFR *molecular pathway**.

At the intracellular scale, the model employs a system of ordinary differential equations (*ODE*s) to describe the intracellular *EGFR *molecular pathway (Figure 2) shown in **equation 1**.

(1) $$\frac{dX_{i}}{dt}=\alpha_{i}\cdot X_{i}-\beta_{j}\cdot X_{j}$$

where *X_i _*is the mass of *ith *molecule of the implemented *EGFR *signaling network, and *α_i _*and *β_i _*are respectively the rates of synthesis and degradation of that molecule. The details of **equation 1 **are listed in Table 1[16].

**Table 1:** [16] - (1) Components of the *EGFR *gene-protein interaction network, (2) Kinetic equations employed to describe the reactions between the *EGFR *species, (3) Coefficients of the *EGFR *gene-protein interaction network taken from the literature

(1)			
**Symbol**	**Molecular variables**	**Initial Condition**	

*X*_0_	*Glucose*	25*mM*	

*X*_1_	*TGFα*	9010.55*nM*	

*X*_2_	*EGFR*	100*nM*	

*X*_3_	*TGFα -EGFR*	0*nM*	

*X*_4_	(*TGFα -EGFR*)^2^	0*nM*	

*X*_5_	*TGFα -EGFR-P*	0*nM*	

*X*_6_	*PLCγ*	10*nM*	

*X*_7_	*TGFα-EGFR-PLCγ*	0*nM*	

*X*_8_	*TGFα-EGFR-PLCγ-P*	0*nM*	

*X*_9_	*PLCγ-P*	0*nM*	

*X*_10_	*PLCγ-P-I*	0*nM*	

**(2)**			

*dX*_1_/*dt=-v*_1_	(1)	*v*_1 = _*k*_1_*X*_1_*X*_2_-*k*_-1_*X*_3_	(11)

*dX*_2_/*dt=-v*_1_	(2)	*v*_2 = _*k*_2_*X*_3_*X*_3_-*k*_-2_*X*_4_	(12)

*dX*_3_/*dt = v*_1_-2*v*_2_	(3)	*v*_3 = _*k*_3_*X*_4_-*k*_-3_*X*_5_	(13)

*dX*_4_/*dt = v*_2_+*v*_4_-*v*_3_	(4)	*v*_4 = _*V*_4_*X*_5_/(*K*_4_+*X*_5_)	(14)

*dX*_5_/*dt = v*_3_+*v*_7_-*v*_4_-*v*_5_	(5)	*v*_5 = _*k*_5_*X*_5_*X*_6_-*k*_-5_*X*_7_	(15)

*dX*_6_/*dt = v*_8_-*v*_5_	(6)	*v*_6 = _*k*_6_*X*_7_-*k*_-6_*X*_8_	(16)

*dX*_7_/*dt = v*_5_-*v*_6_	(7)	*v*_7 = _*k*_7_*X*_8_-*k*_-7_*X*_5_*X*_9_	(17)

*dX*_8_/*dt = v*_6_-*v*_7_	(8)	*v*_8 = _*V*_8_*X*_9_(*K*_8_+*X*_9_)	(18)

*dX*_9_/*dt = v*_7_-*v*_8_-*v*_9_	(9)	*v*_9 = _*k*_9_*X*_9_-*k*_-9_*X*_10_	(19)

*dX*_10_/*dt = v*_9_	(10)		

**(3)**			

Forward rate (*s*^-1^)	Reverse rate (*s*^-1^)	Michaelis constants (nM)	Maximal enzyme rates (nM *s*^-1^)

*k*_1 _= 0.003	*k*_-1 _= 0.06	*K*_4 _= 50	*V*_4 _= 450

*k*_2 _= 0.01	*k_-_*_2 _= 0.1	*K*_8 _= 100	*V*_8 _= 1

*k*_3 _= 1	*k*_-3 _= 0.01		

*K*_5 _= 0.06	*k*_-5 _= 0.2		

*K*_6 _= 1	*k*_-6 _= 0.05		

*K*_7 _= 0.3	*k*_-7 _= 0.006		

*k*_9 _= 1	*k*_-9 _= 0.03		

Giese et al. [19] indicated that a *GBM *cell will not migrate and proliferate at the same time (known as the proliferation-migration dichotomy). In addition, Dittmar et al. [20] reported that a transient increase in phospholipase *Cγ *(*PLCγ*) results in (breast) cancer cell migration. Therefore, we assumed [11,12,15] that once the rate of change of a *GBM *cell's phosphorylated *PLCγ *exceeds the average rate of change of phosphorylated *PLCγ *in cells switching phenotype, the cell becomes migratory; otherwise, it adopts the proliferative phenotype. These conditions for a phenotypic switch are represented by **equation 2**.

(2) $$\{\begin{array}{c} migration,\;if\frac{d\left(PLC\gamma \right)}{dt}>Avg\\ proliferation,\;if\;\frac{d\left(PLC\gamma \right)}{dt}\leq Avg \end{array}$$

where $\frac{d\left(PLC\gamma \right)}{dt}$ denotes the rate of change of phosphorylated *PLCγ *concentration, and *Avg *describes the average rate of change of phosphorylated *PLCγ *of cells switching phenotype at the time step.

### Intercellular scale

A discrete module is employed to simulate a cell's intercellular behaviors. At each time step, a cell will choose the location with highest attraction value to migrate or spawn its off-spring. This process is represented by **equation 3 **[9,15,17,21].

(3) $$T_{ij}=\psi \cdot E_{ij}+\left(1-\psi \right)\cdot \varepsilon_{ij,}$$

where *T_ij _*denotes the attractiveness of location (*i,j*), *E_ij _*is the concentration of *TGFα *at location (*i,j*), and *ε_ij _*~*N*[*μ,σ^2^*] is an error term that is normally distributed with mean *μ *and variance *σ^2^*. The parameter *Ψ *takes on a positive value between zero and one and represents the precision of search. Here we choose *Ψ *= 0.7 on the basis of previous works [9,13,15,17].

### Tissue scale

The chemoattractant diffusion in the tissue is modeled by the diffusion **equation 4**.

(4) $$\frac{\partial Y}{\partial t}=D\cdot \nabla^{2}Y-U+S,$$

where *Y *is the concentration of chemoattractant, *D *is the diffusivity of chemoattractant, *t *is the time step, and *U *and *S *are respectively the cell's chemoattractant uptake and secretion rates.

In general, the multiscale approach incorporates three different scales: intracellular, intercellular and tissue. The intracellular gene-protein interaction pathway affects the intercellular scale by determining a cell's phenotype. In turn, the chemoattractants diffusing at the tissue level affect both the intracellular and tissue scales by stimulating a cell's molecular pathway and remodeling the tumor cells' microenvironment. An important advantage of the multiscale *ABM *approach is that it can be used to analyze and expose the incoherent relations among the different scales. Such analysis may result in experimentally testable hypotheses. However, owing to the complexity of these types of models, real-time simulations of systems with realistic sizes are extremely difficult because forbiddingly huge computation is required. For example, it took approximately seven computing hours on a high performance *CPU *(IBM Bladecenter machine, dual-processor, 32-bit Xeons ranging from 2.8-3.2 GHz, 2.5 GB RAM, and Gigabit Ethernet) to simulate approximately twenty thousand cells (final state) in a 100*100*100 extracellular matrix with relatively coarse grids (around 20 μm) for 20 days [15,17]. Therefore, a realistic *in vitro *tumor simulation with millions of cells on relatively fine grids would require an immense simulation time. To minimize the simulation time and simulate real-time cancer progression, a multi-resolution design [21] was incorporated into the multiscale *ABM*.

### Multi-resolution perspective

A multi-resolution design is used to relieve the huge computational resource demand of *MABM *and visualize tumor progression at various resolutions. In this approach, more computational resource is allocated to heterogeneous regions of the cancer and less to homogeneous regions. In summary, the aim of the multi-resolution approach is to reduce the simulation computing time by sacrificing the accuracy of the simulated results compared with the original *MABM*.

**Figure 3:**
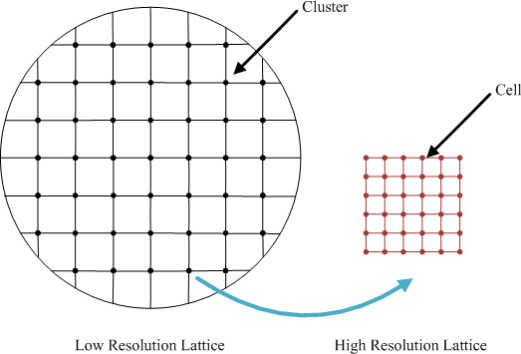
**Configuration of coupled high-resolution and low-resolution lattices**.

To implement the multi-resolution design, a double resolution lattice is developed [16] as in Figure 3. The low resolution lattice size spacing is set to 62.5 μm, which is equal to the smallest unit of the hemocytometer [22] used in experiments. A high-resolution grid with a lattice spacing of 10 μm (approximately equal to a *GBM *cell diameter) is superimposed on the low resolution grid. Here, we define a cell cluster as a group of cells located at a grid point of the low resolution lattice. If cells occupy all the locations of the high resolution lattice affiliated with the grid point of the low resolution lattice, this cell cluster is denoted as a dense cluster.

**Figure 4:**
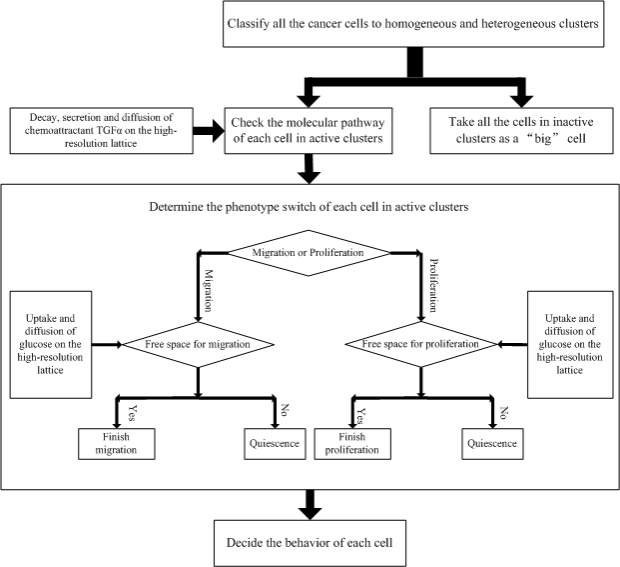
**Multiscale and multi-resolution model**.

Each cancer cell is classified as belong to either a heterogeneous or a homogeneous cluster. Described by Figure 4, only the profile of a cell belonging to a heterogeneous cluster is computed to determine its phenotype switch [16]. Cancer cells in the homogeneous clusters are treated as a single big 'cell'. The classification method is as follows: if all the topographic neighborhoods of a dense cluster are themselves dense, then this cluster is deemed homogeneous; otherwise, it is heterogeneous. Since in the multi-resolution approach the intracellular molecular pathway is computed only for cells belonging to heterogeneous clusters to determine phenotypic switches, the overall computation time required for the simulation is significantly less than in the *MABM*. However, in a realistic cancer progression system, even the heterogeneous clusters of the multi-resolution approach will consist of millions of cells, implying that an enormous computational resource is required to process the cells' intracellular molecular pathways in real time. Furthermore, in order to simulate a realistic cancer progression system, we must employ relatively fine grids to model the tumor's microenvironment. This makes it hard to use current sequential *PDE *solvers to simulate the diffusion of chemoattractants. For these reasons, this study incorporated a *GPU*-based parallel computing algorithm into the multi-resolution *MABM *to accelerate both the *ODE *and *PDE *numerical solvers.

### *GPU*-based parallel computing algorithm

A modern *GPU *is essentially a massively-parallel, explicitly programmable co-processor consisting of hundreds of programmable processors with a natural programming hierarchy [23]. This hierarchy can mimic the bottom-up organization of *ABM *models by setting the intracellular and intercellular scale computations at the bottom (communicating locally via the fast shared memory of the *GPU*) of the hierarchy on the *GPU *while coordinating the logic control module of the model on the *CPU*. Modern *GPU *programming is sufficiently flexible to take advantage of the multi-resolution design by dynamically focusing *GPU *computing resources on the currently heterogeneous regions of the cancer. *Fermi GPUs *(*GTX 480*) have up to 480 processors, which can be bundled together to provide thousands of individual *GPU *processors. This system can provide significant benefits towards scaling feasible *MABM *model computations, which help us to approach the target of simulating realistic tumor growth problems [23]. To speed up the current multi-resolution *MABM*, we parallelized both the chemoattractant diffusion module and the intracellular *EGFR *pathway module.

### Speeding up the computation of the intracellular EGFR molecular pathway module

**Figure 5:**
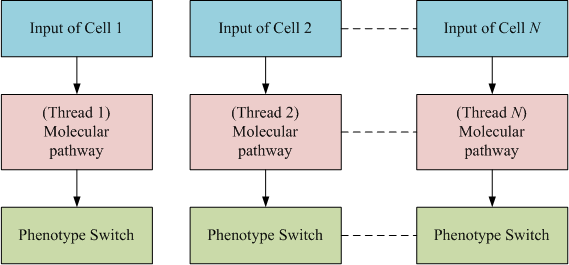
**Parallel *ODE *Solver**.

A *GPU*-based parallel *ODE *solver (Figure 5) was developed to process intensive computing queries from tens of thousands of *GBM *cells during simulations of tumor expansion. For cancer cells in the aforementioned heterogeneous clusters, **equation 1 **is used to determine a phenotypic switch. If we still use a sequential *ODE *solver to process the computation cell by cell in the heterogeneous clusters, it would be impossible to obtain the results in a reasonable time range. For example, it took around 25 seconds to run one step of *ODE *processing for 260 thousand cells with the sequential solver. The *GPU-based ODE *parallel computing algorithm can simultaneously process the computing queries for the cells in the heterogeneous clusters by assigning each cell a thread as shown in Figure 5, which results in a significant increase in the model's performance up to 5.2-fold. In this case, only global memory is employed to accelerate the computation.

### Speeding up the diffusion module

**Figure 6:**
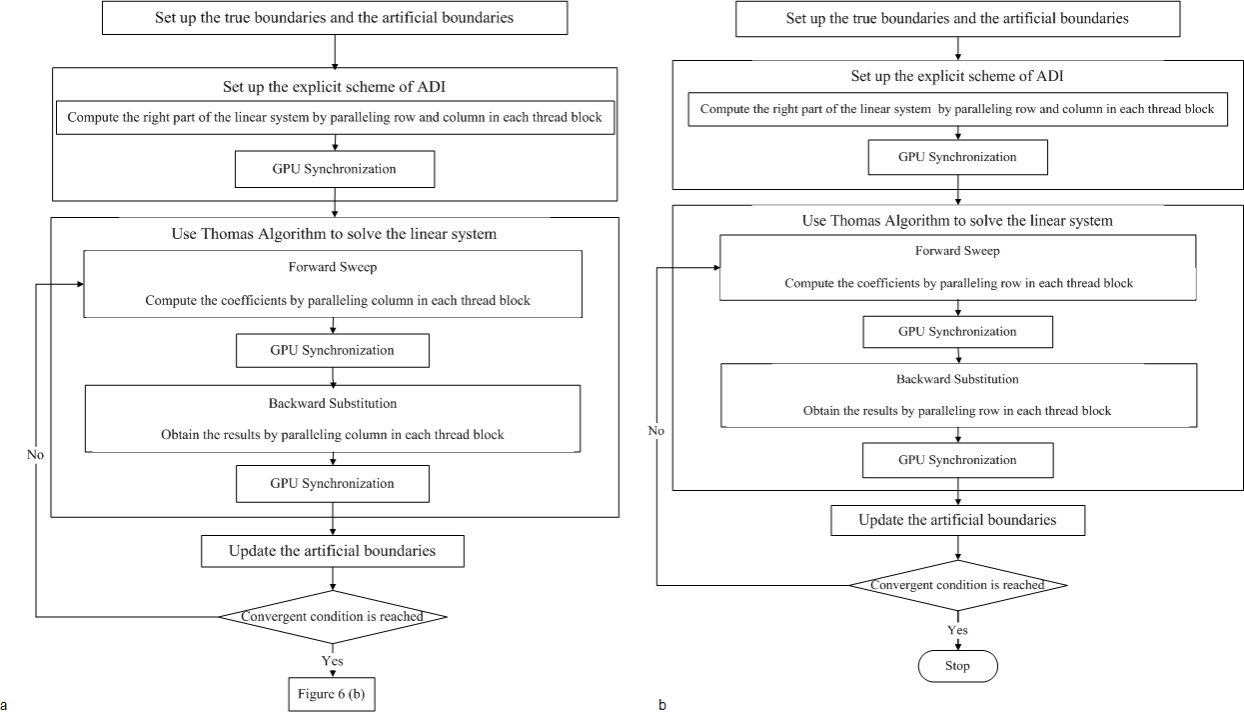
**The flowchart of *PSGMG***.

Previous research [18] has already developed three *GPU-*based parallel algorithms to accelerate the numerical solution of the reaction-diffusion *PDE *equation (**equation 4) **by integrating an alternating direction scheme (*ADI*) [24], Thomas algorithm [24,25] and domain decomposition strategy [26,27], which were incorporated into the new features of *GPU *technology. The first approach is a parallel computing algorithm with global memory (*PGM)*. The second is a parallel computing algorithm with shared memory, global memory and *CPU *synchronization [18,28-30] (*PSGMC*). The third is a parallel computing algorithm using shared memory, global memory and *GPU *synchronization [18,29,31] (*PSGMG*). *PSGMC *and *PSGMG *use a "tiles" strategy to decompose the data and utilize both global memory and shared memory with the classical alternating Schwarz domain-decomposition method [24,26,27,32,33]. Our recent publication [18] demonstrated that *PSGMG *(Figure 6[18]**) **is the fastest parallel algorithm for speeding up the numerical solver of the diffusion equation. Thus, this research employed *PSGMG *to accelerate the diffusion solver of *MABM*.

## Results

Our code was written in Microsoft Visual Studio *C++ *[34,35] and *NVCC *[36] programming languages, We ran the simulation 10 times with different random number seeds (1-100 time steps, one time step being equivalent to one hour) on a Dell workstation with *Fermi GeForce GTX 480 *[37-39] and obtained the average result. The initial condition is described in Table 1.

### Multiscale analysis

**Figure 7:**
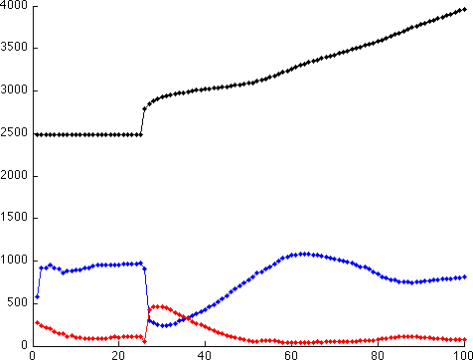
**Population of tumor cells**. The red color represents migratory cells, the blue represents proliferating cells and the black represents all the tumor cells. The *x *axis represents the time step and the *y *axis represents the total number of tumor cells.

**Figure 8:**
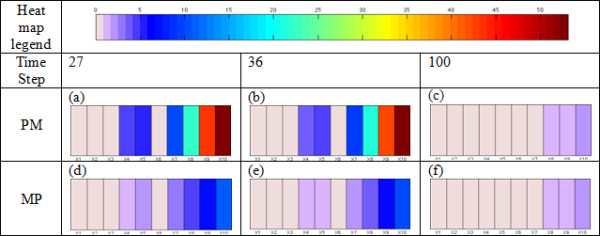
**Molecular profile of *GBM *cells that switch their phenotypes from proliferation to migration at time step t = 27 (a), t = 36 (b) and t = 100 (c)**. **Molecular profile of *GBM *cells that switch their phenotypes from migration to proliferation at time step t = 27 (d), t = 36 (e), and t = 100 (f). **The *x *axis represents the components of the *EGFR *gene-protein interaction network and the average percentage rate of change of each component is represented by heatmaps. (Note: *PM *= phenotypic switch from proliferation to migration; *MP *= phenotypic switch from migration to proliferation).

**Relationship between the tumor cell population and switching molecular profile **Figure 7 describes the population of tumor cells as a function of time, where red, blue and black represent migratory cells, proliferative cells and all the tumor cells, respectively. Since the cell cycle requires several time steps to switch a cell's phenotype, a marked change appears at around *t *= 25 . From Figure 7, we observed that the proliferation curve (blue) crossed the migration curve (red) at *t *= 27 and 36; moreover, both curves became flatter when approaching *t *= 100. As mentioned earlier [15,17], a multiscale analysis can be used to investigate the incoherent relationships between the cells' behaviors (phenotypic switches) and their intracellular molecular profiles. Such an investigation is presented in Figure 8, where we depict the concentrations of different molecules in the *EGFR *network at the three time points mentioned above when phenotypic switches from proliferation to migration or from migration to proliferation occur. In particular, Figure 8(a), (b) and 8(c) show the molecular profiles of cells that switch their phenotypes from proliferation to migration at time points 27, 36 and 100, respectively; Figure 8(d), (e) and 8(f) show the molecular profiles of cells that switch their phenotypes from migration to proliferation at time points 27, 36 and 100, respectively. We infer that the average percentage rates of change of *X*_8 _(*TGFα-EGFR-PLCγ-P*), *X*_9 _(*PLCγ-P*) and *X*_10 _(*PLCγ-P-I*) are larger than the average percentage rates of change of *X*_1_*, X*_2_*, X*_3 _and *X*_6 _(*TGFα*, *EGFR*, *TGFα -EGFR *and *PLCγ*). In the early time stages (time steps 27 and 36), the average percentage rates of change of the molecular species of cells switching their phenotype from proliferation to migration (Figure 8(a) and 8(b)) are significantly greater than the average percentage rates of change of the molecular species of cells switching their phenotype from migration to proliferation (Figure 8(d) and 8(e)). Also, a significant percentage rate of change of *X*_9 _(*PLCγ-P*) resulted in the phenotypic switch. However, the difference between these two molecular profiles (Figure 8(c) and 8(f)) is not as obvious at the final stage (*t *= 100) as in the early stages. In addition, a very trivial percentage rate of change of *X*_9 _(*PLCγ-P*) caused a phenotypic switch.

### Advantages of the multi-resolution approach

#### Visualization of cancer progression at various resolutions

**Figure 9:**
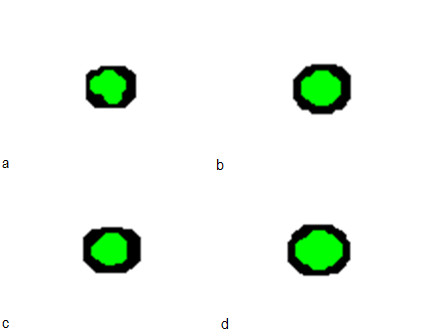
**Tumor progression in the low resolution lattice at time steps *t=*1 (a), *t=*27 (b), *t=*36 (c) and *t=*100 (d)**. The black color represents heterogeneous cell clusters and the green color represents homogeneous cell clusters.

**Figure 10:**
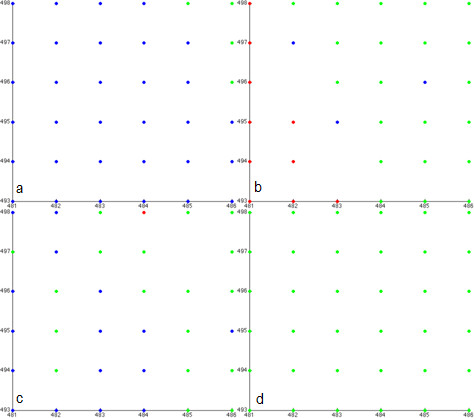
**Cells' behavior on the high-resolution lattice at time steps *t=*1 (a), *t=*27 (b), *t=*36 (c) and *t=*100 (d)**. The red color represents migratory cells, the blue color represents proliferating cells, and green represents quiescent cells. The *x *and *y *axes represent the *x*- and *y*-coordinates of the grid points on the high-resolution lattice, respectively.

**Figure 11:**
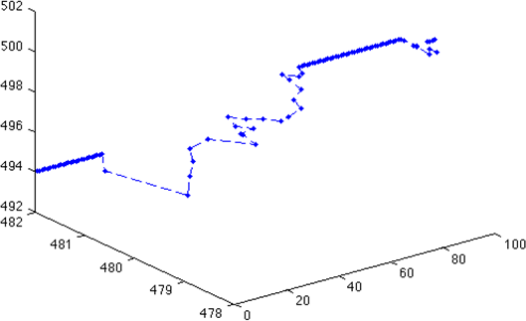
**Trajectory of a single cell**. The *x *axis represents the time step. The *y *and *z *axes represent the *x*- and *y*-coordinates of the grid points on the high-resolution lattice, respectively.

The multi-resolution *MABM *is capable of describing tumor progression at various resolutions. Figure 9 shows tumor progression in the low resolution lattice at time points 1, 27, 36 and 100. The black represents heterogeneous cell clusters and the green represents homogeneous cell clusters. We can already see from Figure 9 that the tumor has a core of homogeneous clusters and a rim of heterogeneous clusters. We can visualize the *GBM *cancer cells' behaviors in the high-resolution lattice at the same time steps. For example, we can choose any cluster and show each cell's phenotype and position in the cluster as shown in Figure 10. Here, red represents migratory cells, blue represents proliferating cells and green represents quiescent cells. Finally, we can track each cell's trajectory as shown in Figure 11, where we show the position of a single cell from time steps 1 to 100.

### Speed-up of the multiscale and multi-resolution *ABM *by *GPU*

#### *GPU*-based *MABM *versus sequential *MABM*

**Figure 12:**
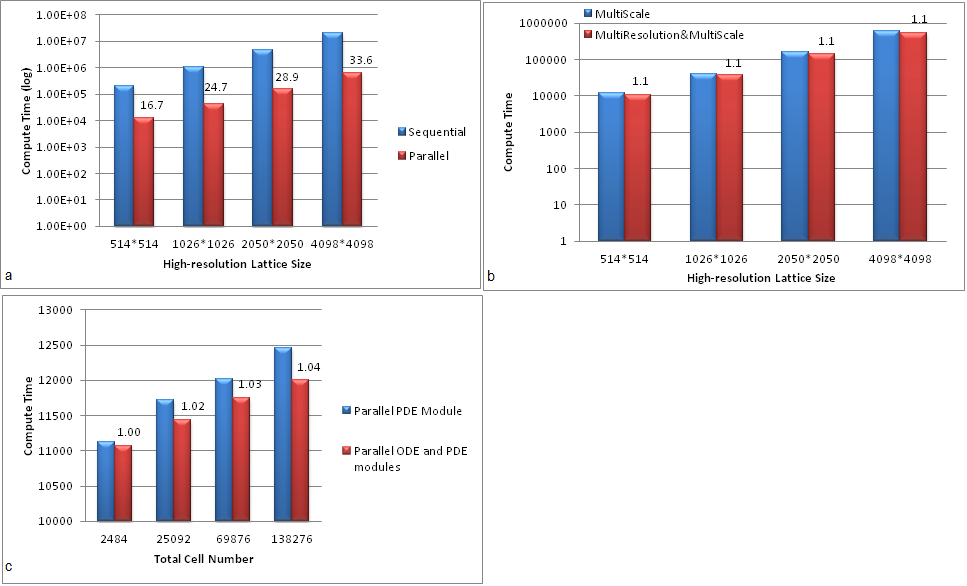
**(a) Computation time of *GPU*-based multiscale model and sequential multiscale model**. The *x*-axis represents the high-resolution lattice size and the *y*-axis represents the computation time (logarithmic scale with base 10) in milliseconds. The blue bar represents the computation time of the sequential multiscale model and the red bar represents the computation time of *GPU*-based multiscale model. The number on each red bar indicates the speed of the parallelized algorithm divided by the speed of the sequential algorithm. **(b) Computation time of *GPU*-based multi-resolution and multiscale model and *GPU*-based multiscale model**. The *x *axis represents the high-resolution lattice size and *y *axis represents the computation time (logarithmic scale with base 10) in milliseconds. The blue bar represents the computation time of *GPU*-based multiscale model and the red bar represents the computation time of the *GPU*-based multiscale and multi-resolution model. The number on the red bar indicates the speed-up of the *GPU*-based multiscale model. **(c) Computation time of *GPU*-based multi-resolution and multiscale model with parallel *ODE *and *PDE *modules and with only parallel *PDE *module**. The *x *axis represents the total cell number and the *y *axis represents the computation time in milliseconds. The blue bar represents the computation time of the *GPU*-based multi-resolution and multi-scale model with only the *PDE *solver parallelized. The red bar represents the computation time of the *GPU*-based multi-resolution and multiscale model with both the *ODE *and *PDE *modules parallelized. The number on the red bar indicates the speed-up of the *GPU*-based multiscale and multi-resolution model with only the *PDE *module parallelized.

Figure 12(a) shows that the *GPU*-based *MABM *is much faster than the sequential *MABM *model. It is clear from this figure that the parallelized code runs at least an order of magnitude faster than the sequential algorithm. In particular, the speedup is markedly increased with respect to the finer grids.

#### *GPU*-based multi-resolution *MABM *versus *GPU*-based *MABM*

The *GPU-*based *MABM *is accelerated further when the multi-resolution design is incorporated into it. Figure 12(b) shows that the *GPU*-based multi-resolution *MABM *has a better performance than the *GPU*-based *MABM*.

#### Speeding up the multi-resolution *MABM *model with a large cell population

As indicated in Figure 12(b), the *GPU-*based parallelized *ODE *solver cannot exhibit its advantage in significantly increasing the performance of the code when the cell population is small, because the diffusion module consumes most of the computational resource. However, Figure 12(c) demonstrates that as the tumor cell number increases on a 514 by 514 high-resolution lattice, the *GPU-*based parallelized *ODE *can significantly increase the performance of the model.

## Discussion and Conclusions

Recently, a variety of cancer research reports have indicated that the *EGFR *pathway plays an important role in the directional motility [40-42], mitogenic signaling [43,44] and phenotypic switching of cancer cells [20,45]. In particular, Dittmar et al. [20] demonstrated that *PLCγ *, a molecular species in the *EGFR *downstream pathway [46,47], is transiently activated in breast cancer cells to a greater extent during migration. In addition, experimental observations of *GBM *suggested that at the same time interval, migrating tumor cells seldom proliferate and proliferating cells seldom migrate [19]. On the basis of these experimental results, Athale et al. [11] assumed that if the percentage rate of change of the phosphorylated *PLCγ *concentration exceeds a pre-specified threshold, *GBM *cells will migrate; otherwise, they will proliferate. Using this assumption, Athale et al. [11,12] and Zhang et al. [15] developed several *in silico *2D and 3D *MABMs *to investigate how perturbations in the intracellular *EGFR *gene-protein network affect the progression of the entire tumor at the intercellular and tissue scales.

However, the above works [11,12,15] were limited by the available computational resources. As indicated by previous research [16], simulating 3D cell growth with an *ABM *model is very time consuming. Scale-up analysis showed that one such simulation would take about 40 days with an IBM Bladecenter machine (dual-processor, 32-bit Xeons ranging from 2.8-3.2 GHz, 2.5 GB *RAM*, and Gigabit Ethernet), which is practically impossible. This limitation prevents simulation using *MABMs *from modeling more realistic large cancer systems. Therefore, the present research incorporated *GPU*-based parallel computing algorithms combined with a multi-resolution design into a multiscale *ABM *to simulate real-time actual *GBM *cancer progression. The *in silico *results demonstrated that our *GPU-*based multi-resolution *MABM *can be used not only to investigate the incoherent relationships among various scales during cancer progression and visualize tumor progression at different resolutions, but also to overcome the computational resource shortage problem and simulate actual cancer progression in real time.

As is well known, computer simulations of complex agent-based systems result in various emergent behaviors due to non-linear interactions among the agents, which in our case are the cancer cells. Similarly, the multiscale analysis of our simulation results revealed various emergent findings. First, the molecular profiles of cells switching phenotypes from proliferation to migration (*PM*) and from migration to proliferation (*MP*) have very similar patterns (Figure 8). Second, we found that *X*_8 _(*TGFα-EGFR-PLCγ-P*) and *X*_10 _(*PLCγ-P-I*) correlated strongly with the rate of change of *X*_9 _(*PLCγ-P*), which determined the cell's phenotypic switch (**Equation 2**), whereas *X*_1 _(*TGFα*)_, _*X*_2 _(*EGFR*)_, _*X*_3 _(*TGFα -EGFR*) and *X*_6 _(*PLCγ*) were independent of the rate of change of *X*_9 _(*PLCγ-P*). Third, at early time stages, a high percentage rate of change of *PLCγ *caused the cell's phenotype to switch from proliferation to migration and a comparatively low percentage rate of change in *PLCγ *caused a switch from migration to proliferation; but the difference in *PLCγ *between these two molecular profiles (*MP *and *PM*) was very small in the final simulation stage. It is noted that the simulation data are from a four day experiment, so we set the simulation duration at 100 hours. These findings imply that the external input (*TGFα*), the major stimulator of the *EGFR *pathway, cannot change the concentration of *PLCγ *substantially at the end stage of simulation.

The multi-resolution design allowed us to visualize the tumor progression at various resolutions. Our simulated results revealed that the heterogeneous clusters consisting of cells with various phenotypes were always on the outer regions of the tumor. In addition, we were able to explore the cells' behavior in the heterogeneous clusters. Using a high resolution lattice we investigated the cells' positions and phenotypes at different time steps. Moreover, the multi-resolution design enabled us to track a cell's trajectory.

We also showed that the performance of the model was significantly improved by employing *GPU*-based parallel computing algorithms. We showed that the parallelized algorithm (*PSGMG*) is much better than the sequential algorithm on large lattices or when the cell population is large.

In summary, the simulation results demonstrated that the *GPU*-based multi-resolution *MABM *has great potential for simulating actual *GBM *tumor progression in real time. In the near future, we plan to incorporate more parameters from experiments into the model, which will enable us to simulate *GBM *progression patterns at various resolutions in a more realistic way. Such simulations will enable us to investigate molecular biomarkers that play an important role in inhibiting cancer expansion and predict real *GBM *progression. Subsequently, we plan to work with experimentalists to use actual data to validate the effectiveness of the model.

## Competing interests

The authors declare that they have no competing interests.

## Authors' contributions

LZ carried out the *MABM *studies, participated in the multi-resolution design and drafted the manuscript. BJ developed the *GPU*-based parallel computing algorithms and drafted the manuscript. YW, CS, PZS, JS and XZ did algorithm development and improved the manuscript. All authors read and approved the final manuscript.
